# Individualised computerised cognitive training for community-dwelling people with mild cognitive impairment: study protocol of a completely virtual, randomised, controlled trial

**DOI:** 10.1186/s13063-022-06152-9

**Published:** 2022-05-05

**Authors:** Stephanie Book, Michael Jank, Anna Pendergrass, Elmar Graessel

**Affiliations:** Centre for Health Services Research in Medicine, Department of Psychiatry and Psychotherapy, Universitätsklinikum Erlangen, Friedrich-Alexander-Universität Erlangen-Nürnberg (FAU), Schwabachanlage 6, 91054 Erlangen, Germany

**Keywords:** Community-dwelling, Computerised cognitive training, Mild cognitive impairment, Non-pharmacological intervention, Randomised controlled trial

## Abstract

**Background:**

People with mild cognitive impairment (MCI) are at increased risk of converting to dementia. Cognitive training can improve the cognitive abilities of people with MCI. Computerised cognitive training (CCT) offers several advantages over traditional paper-and-pencil cognitive training and has the potential to be more individualised by matching task difficulty with individual performance. Recent systematic reviews have reported promising effects of CCT on improving the cognitive capacities of people with MCI. However, the quality of existing studies has been limited, and it is still unclear whether CCT can influence the progression to dementia. We developed an ‘individualised’ CCT (MAKSCog) specialised for people with MCI that automatically matches task difficulty with individual performance and an active control training (‘basic’ CCT). The aims of the present study are (a) to evaluate MAKSCog and (b) to investigate whether it can be applied to maintain the cognitive abilities of people with MCI.

**Methods:**

The present study investigates the effects of CCT on cognition in a randomised controlled intervention study in Germany. Participants are community-dwelling people with a psychometric diagnosis of MCI based on the Montreal Cognitive Assessment (MoCA) and Mini-Mental Status Test (MMSE). Screening and baseline testing are conducted via a videoconferencing assessment and telephone. Participants are randomly allocated. The treatment phase is 6 months with an open phase in which participants can freely decide to continue to use the CCTs. Additionally, both CCTs contain a monthly computerised cognitive assessment that measures different cognitive abilities: information processing speed, memory span, short term memory, and logical reasoning.

**Discussion:**

This is the first study to investigate the effect of MAKSCog, an individualised CCT, specifically developed for people with different subtypes of MCI. A methodological strength is the double-blind, randomised, controlled design and the use of basic CCT as an active control group. The study is conducted entirely virtually with valid telehealth assessments for cognitive function. Methodological limitations might include a restriction to participants who feel comfortable with the use of technology and who own a computer, laptop, or tablet.

**Trial registration:**

ISRCTN ISRCTN14437015. Prospectively registered on 27 February 2020.

## Introduction

Dementia is a challenging disease for healthcare systems around the world [1]. The term mild cognitive impairment (MCI) has been created to describe a transitional state between normal ageing and the early onset of Alzheimer’s disease (AD) [2]. It refers to a state that is defined by the presence of early cognitive impairments that do not yet constitute a dementia syndrome and only very slightly impaired instrumental activities of daily living [3–5]. People with MCI may remain stable, revert back to normal cognition, or progress to dementia [6]. About 15% of people with amnestic MCI in which memory dysfunction is most prominent will progress to having AD annually [7]. In non-amnestic MCI, the impairment of other cognitive features such as language, visuospatial, or executive functions predominates. In the general population, the prevalence of MCI increases with age, at 6.7% for ages 60 to 64 and up to 25.2% for ages 80 to 84 [6].

MCI is the optimal period for intervention so that conversion to AD can be prevented. Pharmacological treatments are not convincing [6], leaving non-pharmacological interventions as the only feasible approach to preventing AD. The available research has focused primarily on physical exercise and cognitive training as the two most significant protective factors for dementia [6, 8]. Still, further research is needed. Cheng et al. [8] concluded that physical exercise and cognitive training might support cognitive functioning in different ways: physical exercise training as ‘hardware’, thus preserving neuronal structural integrity and brain volume, while cognitive training works as ‘software’, thus strengthening the functioning and plasticity of neural circuits.

Cognitive training, traditionally presented as a paper-and-pen exercise, has found its way into the digital world in the form of computer-based tasks that include cognitive exercises, games, and virtual reality. Its emerged popularity can be ascribed to safety, low implementation costs, high availability, and accessibility. Yet another advantage is the potential to provide real-time feedback and more individualised training to participants by adapting task difficulty to individual performance [9]. There is even evidence that computerised training is an effective alternative to paper and pencil cognitive training with comparable or better effect sizes in cognitively healthy community-dwelling older adults [10].

A considerable amount of research evaluating the effects of computerised cognitive training (CCT) for people with MCI has emerged during the last decade (for an overview, see, e.g. [11]). The field is growing rapidly. Previous systematic reviews and meta-analyses of CCT intervention studies have demonstrated promising effects on improving the cognitive capacity of people with MCI [11–16]. For example, across 17 randomised controlled trials (RCT) of moderate quality, Hill et al. [12] reported an overall moderate effect size for cognition (Hedges’ *g* = 0.35) and concluded that CCT is a useful intervention for enhancing cognition in people with MCI. More recently, Zhang et al. [11] reported a small positive effect size for global cognitive function (Hedges’ *g* = 0.23) and moderate positive effect sizes for memory (Hedges’ *g* = 0.30) and working memory (Hedges’ *g* = 0.39). Benefits to mood (depression, anxiety, and apathy) were reported in a meta-analysis that also investigated outcomes in everyday life of cognitive interventions, including CCT in people with MCI [15]. In a recent Cochrane review including eight RCTs, Gates et al. [17] evaluated the effects of at least 12 weeks of CCT on maintaining or improving cognitive functioning and preventing dementia in people with MCI. They applied rigorous quality assessments of the studies. Only very cautiously, they concluded that people with MCI might benefit from CCT in terms of improved cognitive function. Limitations were the very low quality of the evidence and an unclear or high risk of bias in several domains in most of the studies, especially the blinding of participants and personnel. Across all trials, data on incident dementia was missing, which was also claimed by Zhang et al. [11]. Irazoki et al. [18] evaluated 19 studies and provided a qualitative synthesis of the features of different computerised cognitive programs. All of the eleven tools they reviewed had multiple target groups, and there were only two that were specifically marked for people with MCI as the target group.

We developed an easy-to-use software application (MAKSCog for maximum cognition) specifically designed for people with MCI that is available for computer, laptop and tablet and can be used from home. The basis for the development was the non-pharmacological multicomponent MAKS® intervention [19], which has been shown to be an effective treatment for people with MCI and mild and moderate dementia in two independent RCTs [20–22]. The cognitive component of MAKS® was the basis for the development of MAKSCog. What makes MAKSCog special is that the application contains a learning system that automatically chooses exercises that match the person’s level of difficulty, thus allowing for more individualised training. MAKSCog was developed for all subtypes of MCI and addresses different cognitive functions.

The aims of the present study are (a) to evaluate MAKSCog, an individualised CCT for people with MCI and (b) to investigate whether it can be applied to maintain the cognitive abilities of people with MCI. We are comparing MAKSCog with a basic CCT that primarily focuses on leisure activities and serves as an active control condition.

The objective of this paper is to describe the study protocol of our RCT while following the evidence-based reporting guidelines of the SPIRIT Statement [23].

## Methods and analyses

### Objectives

#### Research hypothesis

Individualised CCT (MAKSCog) will lead to statistically significantly greater improvements in cognitive capacities over time in people with MCI as compared with basic CCT (active control group).

#### Exploratory study question

In an open phase of the study, we will investigate long-term cognitive status yearly.

### Study design and setting

A prospective double-blind randomised controlled intervention study is being conducted to test the abovementioned research hypothesis. The overall start date of the study was on 01 April 2019. Recruitment began on 16 March 2020 and will continue until 31 January 2021. The study is being carried out in the metropolitan region of Erlangen/Nürnberg, Bavaria, Germany. At baseline, all study participants are randomly assigned to the intervention (individualised CCT; MAKSCog) or active control group (basic CCT). The reasons for choosing an active control group are as follows: First, since the principal usefulness of CCT is well known [11–16], it would be unethical to use a control group without CCT. Second, by using an active control group, we will be able to evaluate the additional effect of the learning system. After baseline testing (t0), the study participants receive one of the two computerised training applications for their computer, laptop or tablet. It is recommended that they use the training at least 30 min per day 3 days a week during the 6-month intervention phase. Both computerised training applications contain the same computerised cognition assessment that will be delivered and collected once a month (t0–t6). After the end of the 6-month intervention phase, the study participants in both the intervention and control groups will be free to decide whether or not to continue using the computerised training. Then the open phase of the study will begin in which we will examine the exploratory study question. Trial registration data are presented in Table 1.

**Table 1:** Trial registration data

Data category	Information
1. Primary registry and trial identification number	ISRCTN14437015
2. Date of registration in primary registry	27.02.2020
3. Secondary identifying numbers	-
4. Source(s) of monetary or material support	Reinhard Frank-Stiftung
5. Primary sponsor	Reinhard Frank-Stiftung
6. Secondary sponsor(s)	-
7. Contact for public queries	see point 8
8. Contact for scientific queries	Prof. Dr. Elmar Graessel, elmar.graessel@uk-erlangen.de
9. Public title	Dementia prevention for older people suffering from mild cognitive impairment using computerised cognitive training tools
10. Scientific title	Individualised computerised cognitive training for community-dwelling people with mild cognitive impairment
11. Countries of recruitment	Germany
12. Health condition(s) or problem(s) studied	Mild cognitive impairment (MCI)
13. Intervention(s)	Intervention group: Individualised computerised cognitive training, which involves targeted exercises for memory span, information processing, visual-spatial cognition, etc.
	Control group: basic computerised cognitive training, which involves basic exercises for memory span, information processing, visual-spatial cognition, etc.
14. Key inclusion and exclusion criteria	Inclusion criteria: 1. MCI 1.1. Montreal Cognitive Assessment score (MoCA) ≤ 24 1.2. Mini Mental State Examination score (MMSE) ≥ 24 2. Own computer/laptop/tablet and basic skills in their use3. Age ≥ 60 4. Informed consent given
	Exclusion criteria: 1. Completely blind or deaf 2. No personal computer, laptop, or tablet 3. Normal cognition, MoCA ≥ 24 4. Dementia, Mini-Mental Status Examination score < 24 5. Depression, Patient Health Questionnaire 9 score (PHQ-9) ≥ 12 6. Apparent neurological diseases and/or other severe psychiatric diseases
15. Study type	Prospective double-blind randomised controlled intervention study
16. Date of first enrolment	09.06.2020
17. Target sample size	-
18. Recruitment status	incomplete
19. Primary outcome(s)	Cognition measured by the Montreal Cognitive Assessment (MoCA) at baseline and after 6 months
20. Key secondary outcomes	Cognition measured by reaction time and logical thinking assessments delivered and collected by the digital software at baseline and after 1, 2, 3, 4, 5 and 6 months, Mini-Mental Status Examination (MMSE) at baseline and after 6 months Exploratory outcome measures: Long-term cognitive status assessed using the Montreal Cognitive Assessment (MoCA) and the Mini-Mental Status Examination (MMSE), yearly in the open phase

Data are being collected by means of psychometric tests and structured interviews using videoconferencing and telephone. The data ​are being collected by trained student assistants who have no knowledge of group allocation at any time. The procedure is as follows. One of the researchers prepares a folder with the installation application for each study participant on which the software for the assigned computerised training version is located. Shortly before baseline testing (t0), the study participants receive an email with a link to download the software for their version of the computerised application and instructions on how to download and install the software. The participants are not told which of the two applications they are using. At follow-up (t6), after the assessment has been completed, each study participant will also receive an application-specific questionnaire about the usability of the software.

### Sample size and effect size estimation

A power analysis was computed with 100 study participants who were distributed to the two groups of the 2x2 factorial variance-analytic experimental design with one repeated measurement (factor 1: two CCTs; factor 2: two time points). With 50 study participants in each group, alpha = 0.05, beta = 0.20 (corresponding to a power of 80%), a correlation between repeated measures of 0.5 and a nonsphericity correction of 1, we will have the power to detect effects with an effect size of *f* ≥ 0.14 (comparable to Cohen’s *d* ≥ 0.28).

### Recruitment strategies

Participants are being recruited from the general population through individual screening dates. Information channels are media alerts about two newspapers and a magazine for seniors, a regional senior club of retired people, and a health insurance company. The participants are being informed about the project and the planned study in a personal conversation (videoconferencing or phone call).

### Eligibility of participants

Individuals who are interested in the study contact the study centre. We offer them an appointment for screening, including a personal conversation about their screening results afterwards. People who fulfil the criteria for inclusion are informed about the study and asked to take part in the project.

Criteria for inclusion are (1) MCI, psychometrically operationalised by a score on the Montreal Cognitive Assessment (MoCA) ≤ 24 (cut-off for cognitive impairment) and at the same time a score on the Mini-Mental State Examination (MMSE) ≥ 24 (cut-off for no dementia); (2) possession of a computer, laptop, or tablet and basic skills in their use; (3) age ≥ 60; and (4) informed consent. Criteria for exclusion are (1) completely blind or deaf; (2) no personal computer, laptop or tablet; (3) normal cognition, operationalised by a score on the MoCA > 24; (4) dementia, operationalised by a score on the MMSE < 24; (5) acute depression, operationalised by a score on the 9-Item Patient Health Questionnaire (PHQ-9) ≥ 12; or (6) other psychiatric or neurological diseases (checklist): psychosis, (schizophrenia, major depression, mania, bipolar psychosis), Parkinson’s disease, multiple sclerosis, several strokes, alcohol abuse/drug abuse (addiction) and other serious brain diseases (especially brain tumour, brain injury, hydrocephalus). The drop out criteria are (1) withdrawal of consent, (2) serious illness or (3) death of the participant.

The MMSE and the MoCA are administered in combination in order to differentiate between normal cognition, MCI, and dementia. The MoCA is administered first in order to differentiate between normal cognition and MCI on the basis of the cut-off score of 24 points [24]. The MMSE is administered in order to differentiate between MCI and dementia on the basis of the cut-off score of 23 points [25]. For these cut-offs, we looked for an optimised ratio of sensitivity and specificity. The criteria for individuals who were positively screened for MCI, normal cognition, or dementia are shown in Table 2.

**Table 2:** Definition of MCI

	Normal cognition	MCI	Dementia
**Step 1: MoCA**	30-25	24-0	24-0
Step 2: MMSE*	-	30-24	23-0
**Decision**	**Exclusion**	**Inclusion**	**Exclusion**

*Abbreviations*: *MCI* Mild cognitive impairment, *MoCA* Montreal Cognitive Assessment, *MMSE* Mini-Mental State Examination. * MMSE was applied only if the MoCA results were in the range of 24 to 0 points

### Randomisation

Our external biostatistics partner is creating computer-generated randomisation lists (Institute of Medical Informatics, Biometry, and Epidemiology, Friedrich-Alexander University Erlangen-Nürnberg, Waldstraße 6, 91054 Erlangen). All individuals meeting the inclusion criteria are randomised into one of the two groups (individualised CCT or basic CCT). Randomisation is software based and stratified by sex. Couples are assigned to the same group.

### Implementation

Participants do not know which treatment condition they are in, and the student assistants who assess the outcomes of the study are blinded to participants’ allocation.

For each intervention group, a set of download links was created and assigned to the participants in the according group. Similarly, any group-specific test material, e.g. the application-specific usability questionnaire at t6, is assigned by the study team in a sealed envelope.

### Interventions

Both computerised applications (intervention and control) are available for Windows PC/laptop and Android tablet and do not require a connection to the Internet. An Internet connection is needed only to install the application. The participants receive a download link for the respective application and the installation instructions one day before baseline testing. After baseline testing, the student assistants are available to help with the installation. If technical support is needed and the application cannot be installed at baseline, then the start date is set to the day of a successful installation.

#### Individualised CCT for people with MCI (MAKSCog)

The exercises included in this training application have been selected to address the expected level of performance of individuals with MCI. All exercises are available with different levels of difficulty. The playful exercise tasks involve the basic parameters of information processing as well as short-term memory and require different types of decision-making. This application involves ten exercises that focus on different key functions (see Table 3): sustained attention, visuoconstructional reasoning, working memory, recognition memory, visual perception, implicit learning and word finding. These train low to higher cognitive functions. The initial difficulty levels of the exercises are determined by a learning system, which uses (a) a (logistic regression) model that is based on data from people with MCI (individualised by taking into account each participant’s data) and (b) the cognitive status of the participant (i.e. the results of the integrated computerised cognitive assessment) to estimate the likelihood of a participant’s success at a certain difficulty level for a task. The initial model is based on data collected prior to the study: People diagnosed with MCI used the CCT without the learning system. The application chooses the highest level the participant is likely to solve as the entry level. With the learning system, individual (compensation) strategies are nullified, and the ideal level of difficulty for training is generated for each participant.

**Table 3:** Computerised cognitive exercises

CCT application	Group of tasks	Explanation	Key function	Cognitive domain (DSM-5)
**individualised CCT**	Finding targets (‘Punkte sammeln’)	For a set of pop-up pictures, participants must click on target pictures before they disappear	Sustained attention	Complex attention
	Applying rules (‘Regel anwenden’)	Select the winner or loser of a rock-paper-scissors game (either hand signs or in written form); if the game is presented as hand signs, the participant has to pick the winner; if presented with words, the loser has to be picked; this exercise has a time limit, depending on the difficulty	Mental/cognitive flexibility	Executive function
	Layer sorting (‘Ebenen sortieren’)	A target picture of a vase with flowers is presented; the participant has to reproduce the picture out of layers; easiest: background–foreground, up to 5 layers with distractors	Visuoconstructional reasoning	Perceptual-motor
	Jigsaw puzzle (‘Bild zusammensetzen’)	Sorting of image sections	Visuoconstructional reasoning	Perceptual-motor
	Fill in the gaps (‘Felder füllen’)	A grid has to be filled in according to rules; each symbol is used only once in every row, column, and block; layout 4×4 to 9×9 fields	Working memory	Executive functions
	Remember cards (‘Karten merken’)	Remember a row of (up to 5) cards; compare new card to 5th to last card	Working memory	Executive functions
	Find pairs (‘Paare finden’)	Finding pairs of images in a pool; images covered; each turn two cards can be turned	Visuo-spatial memory	Perceptual-motor
	Spot the difference (‘Unterschied erkennen’)	A set of x identical pictures is presented, after a blank, the set and 1 extra pictures are presented; the extra picture has to be selected	Visual perception	Perceptual-motor
	Pattern recognition (‘Schema erkennen’)	A matrix of elements (combination of concentric geometrical figures) is presented; in one row or column, a figure is presented in the same position in all elements; the row/column has to be found; for small difficulties, hints are given	Decision-making	Executive functions
	Word conversion (‘Wörter umwandeln’)	Convert a source word to a target word in *x* steps; in each step, only 1 letter can be exchanged, and each line must contain a word	Word finding	Language
**basic CCT**	Rotating picture puzzle (‘Drehpuzzle’)	Picture is sectioned; sections are rotated; sections have to be turned in the right direction	Visuoconstructional reasoning	Perceptual-motor
	Picture quiz (‘Bilder quiz’)	Multiple-choice questions about images	Semantic and autobiographical long-term memory	Learning and memory
	Geography quiz (‘Länderspiel’)	Knowledge quiz based on German federal states	Semantic and autobiographical long-term memory	Learning and memory
	Dice game (‘Würfelpoker’)	Scoring points in a dice game by rolling five dice to make certain combinations (similar to Yahtzee), playing against a computer	Planning	Executive functions
	Shut the box (‘Klappbox’)	Roll two dice and count the number of dots on each die, sums or differences can be used; the aim is to get all numbers from 1 to 9 once; a player loses when a throw does not derive a new number	Planning	Executive functions

*Abbreviation*: *CCT* computerised cognitive training

#### Basic CCT (active control group)

This training application uses exercise tasks that are oriented towards parlour games and cognitive tasks for everyday life (see Table 3). The exercise tasks, like those of the individualised CCT, are playfully designed and require, among other things, simple strategies, basic arithmetic operations and long-term memory. This application involves five exercises that focus on the following key functions: visuoconstructional reasoning, working memory, semantic and autobiographical long-term memory and planning. Most of the exercises (e.g. the dice game) exist with only a single level of difficulty. The entry-level difficulties of the other exercises are determined solely by the participant’s prior successful results on this exercise. The exercises of the basic CCT aim to provide enjoyable computerised leisure activities with a limited number of cognitive tasks for the active control group.

### Measures

**Fig. 1:**
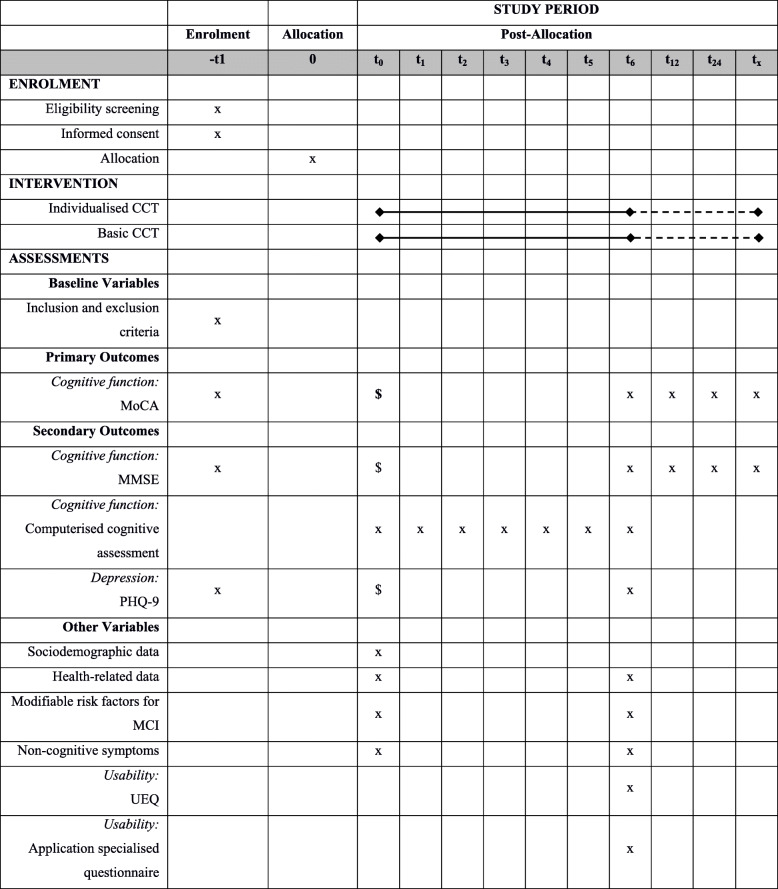
Timeline of measurements. Abbreviations. *CCT* computerised cognitive training, *MCI* mild cognitive impairment, *MoCA* Montreal Cognitive Assessment, *MMSE* Mini-Mental State Examination, *PHQ-9* Patient Health Questionnaire, *UEQ* User Experience Questionnaire. ^$^ The results of −t1 were used, since the timespan between the enrolment test and t0 is maximum 2 weeks

The data are being collected at baseline and follow-up by student assistants (psychology students) trained to conduct performance tests and interviews. The measures that are being used at the different measurement points are shown in Fig. 1.

#### Primary outcome measure

##### Montreal Cognitive Assessment (MoCA) [26]

The MoCA is a performance test that is used to screen for MCI. It consists of more difficult items than the MMSE and is thus able to better detect MCI [26–29]. The score ranges from 0 to 30 points, with higher scores indicating better cognitive performance. A score ≤ 24 indicates cognitive impairment [24]. There are three parallel versions of the MoCA currently available. The MoCA has been found to be an appropriate measure for cognitive screening and has good values for validity and reliability [30].

#### Secondary outcome measures

##### Mini-Mental State Examination (MMSE) [31]

The MMSE is the most frequently employed screening test for dementia [32]. It measures five areas of cognitive functioning: orientation, registration, attention and calculation, recall, and language. The score ranges from 0 to 30 points, with higher scores representing better cognitive performance. Values above 23 points are interpreted as ‘not demented’, whereas scores between 0 and 23 indicate a dementia syndrome [25]. The reliability and validity of the MMSE has been established in numerous studies, e.g. [25, 33, 34].

##### Computerised cognitive assessment

Both versions of the computerised training application contain a set of exercises for measuring different cognitive abilities monthly, beginning at baseline. Eight tests are used to measure various cognitive abilities (see Table 4).

**Table 4:** Computerised cognitive assessment

Test	Description	Adaptation of
Memory span I: Digit span, unsorted (‘Zahlen merken – unsortiert’)	Rows of single-digit numbers are presented (each 1 second); the numbers must be reproduced immediately afterwards	WAIS-IV [35], task Digit Span
Memory span II: Digit span, ascending (‘Zahlen merken – aufsteigend’)	Like Memory span I; numbers must be reproduced in ascending order	WAIS-IV [35], task Digit Span
Processing speed I: Number Comparison (‘Zahlen vergleichen’)	Comparison of two single-digit numbers separated by a horizontal line (participants should react if same number)	Pattern Comparison\Letter Comparison [36]
Processing speed II: Symbol count (‘Symbole zählen’)	Counting a target symbol in a pool as fast as possible	SKT [37], task ‘counting symbols’
Processing speed III: Numerical stroop task (‘numerischer Stroop-Test’)	Two single-digit numbers are presented in different sizes (congruent/incongruent mixed); number with higher value must be clicked as fast as possible	Numerical stroop task [38, 39]
Short term memory I: Free recall (‘Wortliste – Erinnern’)	12 objects have to be named; afterwards shown for 1 minute; some tests later, the objects must be remembered	SKT [37], task ‘delayed recall’
Short term memory II: Cued recall (‘Wortliste – erkennen’)	The objects from Short term memory I must be selected from a selection of 16 objects	SKT [37], task ‘recognition recall’
Logical reasoning: Matrices Test (‘Matrizentest’)	In a (2×2 or 3×3) matrix of symbols, the bottom right symbol is missing; the composition rule has to be understood, and the correct symbol must be selected	Raven Standard Progressive Matrices [40]

*Abbreviations*: *SKT* Syndrom-Kurz-Test (engl. Short Cognitive Perfomance Test), *WAIS-IV* Wechsler Adult Intelligence Scale – Fourth Edition

#### Other variables

##### The 9-Item Patient Health Questionnaire (PHQ-9) [41, 42]

The PHQ-9 is a short self-assessment tool often used in primary care settings to screen for depression [43]. Its nine items cover the nine DSM-IV criteria by asking patients about their experiences during the last 2 weeks and are rated on a four-point scale ranging from 0 (‘not at all’) to 3 (‘nearly every day’). The total sum score suggests varying levels of depression. A cut-off ≥ 12 was found to show a good balance between sensitivity and specificity [44]. The PHQ-9 was found to be a reliable and valid instrument for screening for depression [41].

##### Questionnaire on sociodemographic and health-related data

The following data are being recorded in a standardised questionnaire by the student assistants at baseline: sociodemographic data (age, sex, marital status, highest educational level, employment status, monthly income, household size), modifiable risk factors for MCI (status of general mental activities, physical activities, social participation, sleeping habits, average liquid intake, eating habits, alcohol consumption, nicotine consumption, visual/hearing capacity), non-cognitive symptoms (according to the symptoms of the Neuropsychiatric Inventory [45]) and health-related data (diseases, medications, body weight, body height, dementia cases in family history).

##### User Experience Questionnaire (UEQ) [46]

The UEQ measures attractiveness, perspicuity, efficiency, dependability, stimulation, and novelty of software with 26 bipolar items. The questionnaire consists of pairs of contrasting attributes (e.g. ‘understandable’ vs. ‘not understandable’) that can be rated on a 7-point Likert scale. The UEQ was found to show a satisfactory level of reliability and construct validity [46].

##### Application specialised questionnaire

An application specialised questionnaire is used to identify usability issues. For each exercise, subjective difficulty, specific usability issues, and attractiveness are rated on a 5-point Likert scale. The usefulness and efficiency of the task instructions have to be rated as well.

##### Additional digital data

Both CCTs track usage data. The usage data include the duration of use, difficulty, success, and other parameters for each training task run.

### Data collection

The data are being collected at baseline (t0) and at the end of the 6-month intervention period (t6). As the span between screening and t0 is approximately 2 weeks, we use the MoCA and MMSE of the screening as baseline values. For the exploratory study question, the data are being collected on a yearly basis after the baseline assessment with no specified end point (see Fig. 1). Due to the COVID-19 pandemic, we decided to eliminate the risk of infection related to study participation. The trial will be conducted entirely virtually. All data will be generated via videoconferencing, telephone, or the computerised cognitive assessment that is integrated into the CCTs. The participants were instructed in an invitation mail or letter to prepare an undisturbed environment for the videoconferences. However, participants lacking the hardware for a videoconference or those who are not willing to take part in an assessment by videoconference will be given the option to come into the clinical research centre for their screening visit or follow-up. Testing with the MoCA and MMSE will be conducted via videoconferencing with the student assistants. Videoconferencing assessments with the MoCA and MMSE have very high reliability scores compared with face-to-face testing. The intraclass correlation coefficients (ICC) for the MoCA and the MMSE have been demonstrated in several studies and go up to ICC = 0.99 for the MoCA [47] and up to ICC = 0.92 for the MMSE [48]. The questionnaire on sociodemographic and health-related data will be sent to the study participants to prepare them for the telephone interview. The data collected by the computerised cognitive assessment during the 6-month intervention period will be obtained from the study participants.

### Data quality management

The student assistants involved in the study have been thoroughly trained for their tasks by the study headquarters’ staff. When the study participants have questions concerning the computerised interventions, they can contact the study headquarters by email. The quality of the data is guaranteed by strict data monitoring at the study headquarters for the total study period. Plausibility checks and logical considerations about the relationships between associated variables will be performed. Regular backups will be carried out and saved on an external hard drive.

### Patient and public involvement

Study participants or the public are not involved in the development, design, or conducting of the study. The public has been informed about the recruitment process and the study. In order to recruit study participants from the general population, we provided extensive information about our study through local newspapers, a local magazine for older adults, members of a regional health insurance company, and a regional senior club for retired people. The local newspapers will provide information about the study participants’ experiences with the computerised intervention at the end of the 6-month intervention period.

### Data analysis

All relevant data, sociodemographic, health-related, primary, and secondary outcome variables will be reported descriptively. In order to be able to assess the quality of the randomisation, the baseline data from the intervention and control groups will be tested for statistically significant differences. For the multivariate analyses, we will impute missing values using the expectation maximum algorithm. The primary hypothesis will be tested by calculating multivariate analyses according to the general linear model. To ensure the robustness of the results, we will perform both analysis strategies ‘intention to treat’ and ‘per protocol’. ‘Intention to treat’ evaluations are carried out with all cases still alive at the end of the intervention or observation period. The significance level is defined as alpha = 0.05. The data analyses will be performed using the ‘IBM SPSS Statistics 24’ software. The members of the data monitoring committee are Prof. Dr. Olaf Gefeller and one of his statistics experts from the Institute of Medical Informatics, Biometry, and Epidemiology, Friedrich-Alexander Universität Erlangen-Nürnberg, Waldstraße 6, 91054 Erlangen.

### Ethical considerations

All legal matters such as voluntariness, right of revocation, and General Data Protection Regulation (European Union) are taken into account. People with MCI are independent and fully capable of conducting business and giving consent. Upon agreement, consent to participate (written informed consent) will be obtained from all participants by the student assistants who are members of the study centre. All participants are informed about the study in a personal videoconference after they are screened for eligibility. A participant information sheet including important information about participation (e.g. randomisation, data protection, data storage) is given to every participant. The opportunity to ask questions will be granted by videoconference, telephone, and email afterwards at any time. Participants are not being given any financial inducement to participate.

The external Reinhard Frank-Stiftung is continuously being informed about the progress of the study. In the case of important protocol modifications, we will inform the Ethics Committee, the funder, and the platform for the trial registry.

### Data handling

The informed consent sheet and other personal data are stored in a locked steel cabinet. Only members of the study team have access to the lists of participants’ names and codes. All data are stored in only a pseudonymised form digitally on a firewall-secured server at the University Hospital Erlangen. In order to be able to clearly assign data from different study participants to the previous data records over the course of a longitudinal study, a key file containing the connection between real names and pseudonyms is necessary. The key file is stored on an offline computer and in printed form in a locked metal cabinet. Access to the room (electronic lock) with the metal cabinet is only possible for a limited and precisely defined number of employees of the University Hospital. Access to the key file stored in the metal cabinet is only available to project employees who are obliged to maintain secrecy towards third parties through their employment contract. A transfer of data to third parties (e.g. other research groups) is not planned because it was not included in the information sheet. Results of the study for scientific or other publications are only published in aggregate form (mean values, etc.). No published material will contain patient-identifying information.

### Safety considerations

The CCT applications might have an impact on existing excessive computer use. However, both CCT applications that we developed are not based on motivational or emotional components. The CCT applications require cognitive performance, which could instead lead to exhaustion.

## Discussion

In this paper, we describe the design of a virtual, double-blind RCT for evaluating an individualised CCT (MAKSCog) for people with MCI. We further describe how we will investigate whether it can be applied to maintain the cognitive abilities of people with MCI. This is the first study in which we present MAKSCog, which was specifically designed for the needs of people with different subtypes of MCI.

### Individualised computerised cognitive training (MAKSCog)

MAKSCog addresses several cognitive functions and can therefore be used by people with different subtypes of MCI. A key part of MAKSCog is the automated adaptation of difficulty by machine learning (ML). MAKSCog aims to improve the beneficial effects of CCT by providing exercises at the difficulty level that fits each participant best. Because exercises that are at a person’s skill level are challenging but not overwhelming, the impact of the training can be increased, and the adherence to MAKSCog can be optimised. The ML-System determines the highest starting difficulty level at which the participant is likely to succeed on the basis of the participant’s current cognitive status (i.e. the most current results of the digital cognitive tests) and the results of previous exercise runs. This level is chosen to grant the participants success and to increase their motivation for training. After the successful execution of some rounds, the difficulty is increased, and the participants are trained at their peak. By adding personal results to the ML-System, individual strategies (or a lack thereof) can be eliminated as a factor of success in the exercises, and solely the person’s cognitive abilities determine how well the difficulty of the exercises fit. The ML-System of MAKSCog was pre-trained with usage data collected prior to this study.

### Strengths and limitations of the study design

The strengths of the study design are the randomisation, the double-blinding, the active control group, and the longitudinal character of the study with a long follow-up period of 6 months and an open phase in which study participants will be assessed once a year.

The trial will be conducted entirely virtually as a consequence of the COVID-19 pandemic. This has several implications. First, measurements are limited to those that can be administered by videoconferencing. Larger neuropsychological test batteries cannot be applied. We diagnose participants ‘psychometrically’ by combining the MoCA and the MMSE. This is not adequate for a clinical diagnosis of MCI. We will take this into account when interpreting the results. However, there is ample evidence that the MoCA and the MMSE are reliable and valid screening measures [25, 30, 33, 34, 47, 48]. In a recent systematic review [49], the MoCA and the MMSE were described as valid telehealth measures for screening cognitive status. Indeed, telemedicine is an emerging new field, and there is evidence that it is a valuable tool for assessing neurodegenerative diseases [49–51]. Second, this might lead to a limitation of its use to only participants who feel comfortable using technology or have family members or friends who can assist them. Additionally, a smartphone, laptop, tablet, or computer with microphone and camera is necessary for the virtual assessments. The study is therefore limited to people who have this technical equipment. On the other hand, it is an advantage of this procedure that people who live far from the study centre can also participate.

## Trial status

Protocol version 1.0, 14 February 2020. The overall start date of the study was 01 April 2019. Recruitment began on 16 March 2020 and is expected to be completed until 31 January 2021.

### Protocol amendments

Originally, the screening tests as well as the evaluations at t0 and t6 were planned to be carried out by our student assistants in person. Due to the COVID-19 pandemic, we decided to eliminate the risk of infection related to study participation and changed the conduction of the trial into entirely virtually before the enrolment of the first participants.

## Data Availability

On completion of this study, the final datasets used and/or analysed during the current study will be available from the corresponding author on reasonable request.

## Abbreviations

AD: Alzheimer’s disease
CCT: Computerised cognitive assessment
ICC: Intraclass correlation coefficients
MAKSCog: An individualised CCT specifically designed for people with MCI on the basis of the cognitive component of the MAKS® intervention
MCI: Mild cognitive impairment
ML: Machine learning
MMSE: Mini-Mental State Examination
MoCA: Montreal Cognitive Assessment
PHQ-9: 9-Item Patient Health Questionnaire
RCT: Randomised controlled trial
SKT: Syndrom-Kurz-Test (engl. Short Cognitive Performance Test)
UEQ: User Experience Questionnaire
WAIS-IV: Wechsler Adult Intelligence Scale – Fourth Edition
